# Association between red cell distribution width and all-cause mortality in patients with breast cancer: A retrospective analysis using MIMIC-IV 2.0

**DOI:** 10.1371/journal.pone.0302414

**Published:** 2024-05-15

**Authors:** Jie Xiao, Libi Tan, Yujie Pei, Ruifang Yang, Jing Li, Yong Feng, Jing Feng

**Affiliations:** 1 Department of Pathology, Longhua Hospital, Shanghai University of Traditional Chinese Medicine, Shanghai, China; 2 Department of Laboratory Medicine & Central Laboratory, Southern Medical University Affiliated Fengxian Hospital, Shanghai, China; 3 School of Laboratory Medicine and Biotechnology, Southern Medical University, Guangzhou, China; 4 Anhui University of Science and Technology Affiliated Fengxian Hospital, Shanghai, China; 5 General Surgery, Shiyan People’s Hospital, Shiyan, China; 6 The Second Affiliated Hospital, The Chinese University of Hong Kong, Shenzhen, China; University of Montenegro-Faculty of Medicine, MONTENEGRO

## Abstract

**Purpose:**

Investigating the association between red cell distribution width (RDW) and all-cause mortality in patients with breast cancer, to evaluate the potential clinical prognostic value of RDW.

**Methods:**

Based on the RDW index, patients with breast cancer in the Medical Information Mart for Intensive Care (MIMIC-IV) database were categorized into quartiles. The primary outcomes included in-hospital mortality from all causes during the first six months, the first year, and the first three years. Cox hazards regression and restricted cubic spline (RCS) models were developed to investigate the effects of RDW on primary outcomes.

**Results:**

The study included 939 patients (female). The 6-month, 1-year, and 3-year mortality rates were 14.0%, 21.4%, and 28.4%, respectively. Multivariate Cox proportional hazards analyses demonstrated that RDW exhibited an autonomous association with an increased risk of all-cause mortality. After adjusting for confounders, higher RDW quartiles were significantly associated with 6-month mortality (adjusted hazard ratio (HR), 3.197; 95% confidence interval (CI), 1.745–5.762; *P* < 0.001), 1-year mortality (adjusted HR, 2.978; 95% CI, 1.867–4.748; *P* < 0.001), and 3-year mortality (adjusted HR, 2.526; 95% CI, 1.701–3.750; *P* < 0.001). The RCS curves demonstrated that high RDW (> 14.6) was associated with a greater risk of all-cause mortality. Subgroup analyses revealed no statistically significant differences in the interactions between the subgroups.

**Conclusion:**

The study revealed a highly pronounced relationship between RDW and overall mortality, indicating its potential as an autonomous prognostic factor for increased mortality among patients with breast cancer.

## Introduction

Breast cancer is a common cancer affecting women worldwide. The American Cancer Society predicts that breast cancer will contribute to approximately 32% of newly diagnosed cases in women by 2024 [1]. Despite the continuous progress in medical innovations that have led to a gradual reduction in mortality rates, breast cancer remains the foremost contributor to cancer-related fatalities in the female population [2]. Consequently, it is imperative to employ efficient screening techniques to accurately evaluate the risk of mortality in the clinical diagnosis of breast cancer, as this greatly influences treatment decisions and patients’ clinical outcomes. In the assessment of breast cancer, the commonly utilized prognostic factors include age, tumor size, axillary lymph node status, and histological characteristics (particularly histological grade and lymphatic invasion) [3]. Additionally, molecular subtypes including human epidermal growth factor receptor 2 (HER2) [4], estrogen receptor (ER) [5], progesterone receptor (PR) [6], and antigen Ki-67 [7] are considered important for prognostics evaluation. Currently, the identification of histological features and molecular subtypes relies upon pathological biopsies, which are invasive, time-consuming, and relatively expensive, limiting their widespread clinical use. Consequently, there is a crucial need to explore alternative indicators such as routine complete blood counts, which can provide quick and straightforward insights to assist clinicians in determining the prognosis of patients with breast cancer.

The red cell distribution width (RDW) is a straightforward hematologic parameter that represents the heterogeneity of red blood cell volume [8]. The higher the RDW value, the greater the change in red blood cell size. Fluctuations in RDW have been reported in many pathophysiological conditions; for example, elevated RDW is associated with acute and chronic heart failure [9], coronary artery disease, cerebral infarction, and acute myocardial infarction [10]. Previous studies have reported an association between elevated RDW levels in elderly patients and unfavorable outcomes in terms of overall survival (OS) and disease-free survival (DFS) [11]. Furthermore, based on data from a pilot study, Seretis et al. suggested that RDW may serve as a potential biomarker of breast cancer activity [12]. However, research on the role of RDW in breast cancer prognosis is limited, particularly large-scale studies. Takeuchi et al. analyzed 299 patients with breast cancer and found no significant association between RDW and DFS [13]. In contrast, Huang et al. identified RDW as a relevant inflammatory marker in patients with breast cancer, potentially associated with DFS and OS in young females [14]. Additionally, Yoo et al. demonstrated that preoperative elevation of RDW (>13.5) in patients with breast cancer has the strongest predictive ability for postoperative mortality, with the risk of recurrence and death increasing by approximately 1.7 times once RDW exceeds the critical threshold [15]. In the existing body of research, there are divergent opinions among researchers regarding the impact of RDW on breast cancer patients. In contrast, our study selected a cohort of breast cancer patients across all age groups from the Medical Information Mart for Intensive Care (MIMIC-IV) database. We adjusted for a series of confounding variables and further stratified the patients into specific subgroups to validate the robustness of the analytical results. We aimed to investigate the potential relationship between RDW and overall mortality in patients with breast cancer, elucidating the precise role of RDW in the prognosis of breast cancer.

## Material and methods

### Study population

**Fig 1:**
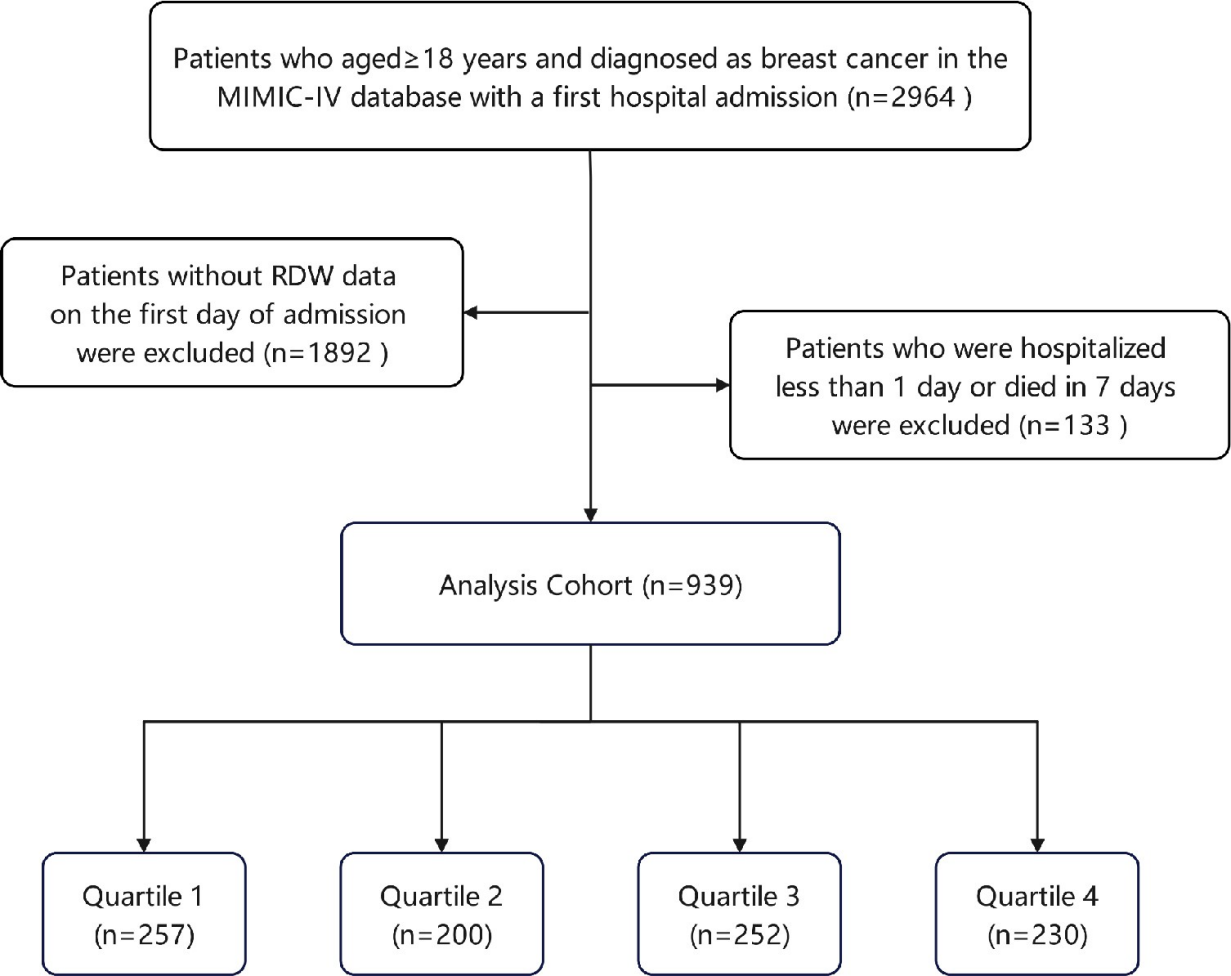
Flow of included patients through the trial. Abbreviations: MIMIC-IV, the medical information mart for intensive care IV; RDW: red cell distribution width.

This retrospective study investigated health-related data from the MIMIC-IV database version 2.0, a comprehensive and extensive single-center database administered by the Laboratory of Computational Physiology at the Massachusetts Institute of Technology (MIT). The MIMIC-IV database is a valuable resource that offers a substantial collection of meticulously documented medical records encompassing resident patients at the Beth Israel Deaconess Medical Center in Boston from 2008 to 2019, relating specifically to patients admitted to the intensive care unit (ICU) [16]. Access to MIMIC-IV requires passing the Protecting Human Research Participants online course and exams from the National Institute of Health. Data extraction was mainly completed by one of the authors (Jie Xiao) after obtaining access to the datasets (certification number: 56775311). The MIMIC-IV database was approved for research by the Institutional Review Boards of the Massachusetts Institute of Technology and Beth Israel Deaconess Medical Center (BIDMC); thus, this study adhered to the ethical standards outlined in the Helsinki Declaration and received a waiver of ethical approval and informed consent. Patients with breast cancer were selected from the MIMIC-IV database based on the diagnostic criteria outlined in the 9th and 10th editions of the International Classification of Diseases. For patients admitted to the ICU multiple times, only the initial admission data were analyzed. The exclusion criteria for this study were as follows: (1) individuals under the age of 18 years at initial admission; (2) patients with incomplete RDW information recorded on the first day of admission; (3) hospitalization within 1 day; (4) death within 7 days of hospitalization. Finally, a comprehensive sample of 939 participants was enrolled and subsequently allocated to four distinct groups based on the RDW quartile (Fig 1).

### Data collection

Baseline characteristics were extracted from the MIMIC-IV database using the PostgreSQL (version 15.3) and Navicat Premium (version 16) software. The categorization of accessible variables was segregated into four distinct groups as follows: (1) demographic information encompassing age, race, sex, stature, mass, and body mass index (BMI); (2) treatment procedures, such as radiation, medication, and breast surgery; (3) comorbidities, including myocardial infarction (MI), congestive heart failure (CHF), peripheral vascular disease (PVD), cerebrovascular disease (CVD), chronic pulmonary disease, rheumatic disease, mild liver disease, and renal disease; (4) laboratory indicators, including anion gap (AG), bicarbonate, white blood cells (WBC), red blood cells (RBC), platelet (PLT), hemoglobin (Hb), hematocrit (HCT), chloride, serum calcium, serum potassium, serum sodium, glucose (GLU), serum creatinine, blood urea nitrogen (BUN), mean corpuscular hemoglobin (MCH), mean corpuscular volume (MCV), mean corpuscular hemoglobin concentration (MCHC), and RDW. The value of the RDW index was obtained as the (standard deviation [SD] of red cell volume/ MCV) ×100 [17]. Data extracted from the ICU included laboratory variables reported within the initial 24-hour period of patient admission. Subsequent analyses commenced on the day of admission and continued until the day of mortal demise. Owing to the occurrence of missing data in MIMIC-IV, a single imputation approach was employed to address any gaps. To circumvent the potential bias resulting from directly inputting missing values, variables displaying a missing rate exceeding 20% were transformed into dummy variables within the models. Additionally, variables with >25% missing values were excluded (S1 Table).

### Statistical analysis

The study population was divided into four groups based on the quartiles of the RDW index on the first day of ICU admission. Mean ±SD or median with interquartile range were used to express continuous variables, while frequency and percentage (%) were used for categorical variables. The normality of continuous parameters was evaluated using the Kolmogorov-Smirnov test. When a normal distribution was met, a t-test or ANOVA was used for comparison. When a non-normal distribution was encountered, the Mann-Whitney U test or Kruskal-Wallis test was used. Fisher’s exact test or Pearson’s chi-square test was used to examine the differences between groups for categorical variables. The incidence of primary end-events (6-month mortality, 1-year mortality, and 3-year mortality) was examined using Kaplan-Meier survival analysis in groups with varying RDW index values (1 unit and quartile), and disparities between groups were assessed using log-rank tests. To determine the hazard ratio (HR) and 95% confidence interval (CI) between RDW and primary endpoints, Cox proportional hazards models were employed, with certain models being adjusted. Baseline variables were used as candidate predictors in the multiple regression models. To avoid model overfitting, we calculated the variance inflation factor (VIF) to quantify multicollinearity between variables and removed any variables that VIF ≥5. Ultimately, only clinically significant and prognostically impactful confounding factors were included in the multivariate model as follows: Model 1, unadjusted; Model 2, adjusted for age, race, and BMI; and Model 3, adjusted for age, race, BMI, radiation, medication, breast surgery, MI, CHF, PVD, CVD, chronic pulmonary disease, rheumatic disease, mild liver disease, and renal disease. To examine the non-linear relationship between RDW and all-cause mortality, we used a restricted cubic spline (RCS) regression model with four knots. Additionally, we employed Receiver Operating Characteristic (ROC) curves to establish the optimal threshold for RDW. In our analysis, the RDW index was included in the model as a continuous variable, and alternatively as a categorical variable. The lowest RDW quartile served as the reference group for the latter approach. To assess any potential trends, P-values were determined based on quartile levels. Moreover, we conducted additional stratified analyses considering age (≥65 years and < 65 years), race (white and non-white), BMI (< 30 kg/m^2^ and ≥ 30 kg/m^2^), diabetes, and renal disease. The objective was to evaluate the consistent prognostic significance of the RDW index for the primary endpoints using likelihood ratio tests to examine any interactions between RDW and the stratified variables. All data analyses were conducted using the SPSS Statistical Software (IBM SPSS Statistics, version 29.0), Prism software (GraphPad Prism, version 9.4.0), and R software (R, version 4.3.6). A double-sided *P* < 0.05 was considered statistically significant.

## Results

This study included 939 patients diagnosed with breast cancer; with an average age of 64.95 ± 14.05 years. All participants were females, with 657 (70.0%) identifying as white. The median RDW index for the entire population stood at 14.6, with an interquartile range (IQR) between 13.4 and 16.4. At a mean follow-up duration of 26.3 months, 350 (37.3%) patients died from any cause. The mortality rates at 6 months, 1 year, and 3 years were 14.0%, 21.4%, and 28.4%, respectively (Table 1).

**Table 1:** Baseline characteristics of critical patients with BC grouped according to RDW index quartiles.

Categories	Overall (N = 939)	Q1 (N = 257)	Q2 (N = 200)	Q3 (N = 252)	Q4 (N = 230)	*p*-value
Age, years	64.95 (14.05)	64.31 (13.74)	65.49 (15.46)	65.94 (14.49)	64.10 (12.55)	0.403
BMI, kg/m^2^	27.94 (23.83, 33.25)	25.85 (22.98,30.64)	28.14 (24.48, 33.92)	28.65 (24.36, 33.95)	28.72 (24.61, 34.61)	<0.01
White, n (%)	657 (70.0)	198 (77.0)	139 (69.5)	170 (67.5)	150 (65.2)	0.025
Radiation, n (%)	37 (3.9)	9 (3.5)	3 (1.5)	12 (4.8)	13 (5.7)	0.138
Medication, n (%)	13 (1.4)	2 (0.8)	1 (0.5)	4 (1.6)	6 (2.6)	0.220
Breast Surgery, n (%)	131 (14.0)	61 (23.7)	41 (20.5)	23 (9.1)	6 (2.6)	<0.001
Comorbidities, n (%)						
Myocardial infarction	44 (4.7)	9 (3.5)	9 (4.5)	15 (6.0)	11 (4.8)	0.63
Congestive heart failure	109 (11.6)	12 (4.7)	25 (12.5)	39 (15.5)	33 (14.3)	<0.001
Peripheral vascular disease	28 (3.0)	7 (2.7)	2 (1.0)	11 (4.4)	8 (3.5)	0.2
Cerebrovascular disease	50 (5.3)	17 (6.6)	9 (4.5)	18 (7.1)	6 (2.6)	0.105
Chronic pulmonary disease	170 (18.1)	35 (13.6)	30 (15.0)	58 (23.0)	47 (20.4)	0.021
Rheumatic disease	26 (2.8)	5 (1.9)	4 (2.0)	5 (2.0)	12 (5.2)	0.079
Mild liver disease	59 (6.3)	14 (5.4)	8 (4.0)	21 (8.3)	16 (7.0)	0.256
Diabetes without CC	147 (15.7)	28 (10.9)	36 (18.0)	43 (17.1)	40 (17.4)	0.105
Diabetes with CC	53 (5.6)	7 (2.7)	8 (4.0)	28 (11.1)	10 (4.3)	<0.001
Renal disease	95 (10.1)	16 (6.2)	14 (7.0)	42 (16.7)	23 (10.0)	<0.001
Severe liver disease	28 (3.0)	2 (0.8)	2 (1.0)	8 (3.2)	16 (7.0)	<0.001
Metastatic solid tumor	399 (42.5)	83 (32.3)	61 (30.5)	112 (44.4)	143 (62.2)	<0.001
Laboratory tests						
AG, mmol/L	13.73 (12.00, 15.00)	13.73 (12.00, 15.00)	13.73 (12.00, 15.00)	14.00 (12.00, 16.00)	13.00 (12.00, 15.00)	0.066
Bicarbonate, mmol/L	25.01 (23.00,27.00)	25.01 (24.00, 27.00)	25.01 (24.00, 27.00)	25.00 (23.00, 27.00)	25.00 (23.00, 27.00)	0.038
WBC, ×10^9^/L	7.40 (4.90, 10.80)	7.80 (5.30, 10.55)	8.25 (5.70, 11.75)	7.20 (4.70, 10.68)	6.65 (4.20, 11.00)	0.037
RBC, ×10^12^/L	3.46 (3.05, 3.92)	3.66 (3.30, 4.07)	3.71 (3.28, 4.09)	3.36 (2.93, 3.80)	3.14 (2.79, 3.60)	<0.001
PLT, ×10^9^/L	218 (162, 285)	211 (170, 265)	222 (174, 269.75)	224.5 (160.25, 302.75)	214 (136, 297.25)	0.351
Hb, g/dL	10.3 (9.0, 11.6)	11.1 (10.2, 12.3)	10.9 (9.73, 12.2)	9.9 (8.9, 10.9)	9.0 (80, 10.1)	<0.001
HCT, %	31.40 (27.70, 35.40)	33.70 (30.25, 37.10)	32.80 (29.73, 36.90)	30.50 (27.43, 33.80)	28.05 (25.18, 31.35)	<0.001
Chloride, mmol/L	103.0 (100.0, 105.0)	102.7 (101.0, 105.0)	103.0 (102.0, 105.0)	103.0 (100.0, 106.0)	103.0 (99.0, 105.0)	0.436
Calcium, mmol/L	8.68 (8.30, 9.00)	8.68 (8.40, 8.90)	8.68 (8.50, 9.10)	8.68 (8.22, 9.00)	8.68 (8.10, 8.90)	0.018
Potassium, mmol/L	4.01 (3.70, 4.20)	4.01 (3.80, 4.20)	4.01 (3.80, 4.20)	4.00 (3.60, 4.30)	4.00 (3.60, 4.30)	0.248
Sodium, mmol/L	138.6 (137.0, 141.0)	138.6 (137.0, 141.0)	139 (138.0, 141.0)	139 (137.0, 141.8)	138.0 (136.0, 140.0)	0.003
GLU, mg/dL	115.0 (95.0, 131.0)	118.0 (96.5, 123.5)	123.0 (99.3, 136.0)	115.5 (92.3, 138.0)	107.0 (90.8, 131.0)	0.016
Creatinine, mg/dL	0.80 (0.60, 0.95)	0.80 (0.60, 0.95)	0.80 (0.63, 0.95)	0.80 (0.60, 1.10)	0.70 (0.60, 1.00)	0.036
BUN, mg/dL	15.00 (10.00, 18.00)	14.00 (10.00, 17.23)	17.00 (11.00, 17.23)	16.00 (11.00, 23.00)	14.00 (9.00, 20.00)	0.023
MCH, pg	30.0 (28.2, 31.3)	30.5 (29.4, 31.8)	30.0 (28.7, 31.1)	29.7 (27.9, 31.2)	29.1 (26.0, 31.0)	<0.001
MCHC, %	32.9 (31.8, 33.7)	33.3 (32.5, 34.2)	33.0 (32.1, 33.7)	32.6 (31.6, 33.5)	32.1 (30.8, 33.5)	<0.001
MCV, fL	91 (87, 95)	92 (88, 95)	90 (87, 94)	91 (86, 96)	90 (83, 95)	0.008
RDW index, %	14.6 (13.4, 16.4)	12.9 (12.5, 13.2)	13.9 (13.7, 14.2)	15.2 (14.8, 15.8)	18.0 (17.1, 19.9)	<0.001
Events						
LOS Hospital, days	4.08 (2.29, 6.63)	3.75 (2.08, 5.31)	3.67, (2.08, 5.54)	4.29 (2.63, 7.54)	4.92(2.79, 7.79)	<0.001
6-month mortality, n (%)	131 (14.0)	15 (5.8)	16 (8.0)	39 (15.5)	61 (26.5)	<0.001
1-year mortality, n (%)	201 (21.4)	25 (9.7)	26 (13.0)	63 (25.0)	87 (37.8)	<0.001
3-year mortality, n (%)	267 (28.4)	37 (14.4)	35 (17.5)	92 (36.5)	103 (44.8)	<0.001

RDW index: Q1 (10.8–13.4), Q2 (13.4–14.6), Q3 (14.6–16.4), Q4 (16.4–26.8)

Abbreviations: BC, breast cancer, RDW, red blood cell distribution width; BMI, body mass index; CC; complications or comorbidities; AG, anion gap; WBC, white blood cell; RBC, red blood cell; PLT, platelet; Hb, hemoglobin; HCT, hematocrit; GLU, glucose; BUN, blood urea nitrogen; MCH, mean corpuscular hemoglobin; MCHC, mean corpuscular hemoglobin concentration; MCV, mean corpuscular volume; LOS, length of stay.

### Baseline characteristics

Table 1 presents the baseline characteristics of patients with breast cancer based on RDW quartiles. The participants were divided into four distinct groups [quartile (Q)1: 10.8–13.4; Q2: 13.4–14.6; Q3: 14.6–16.4; Q4: 16.4–26.8] according to the level of admission RDW. The median RDW value of each group ranged from 12.9 (IQR: 12.5, 13.2) to 13.9 (IQR: 13.8, 14.2) to 15.2 (IQR: 14.8, 15.8), and 18.0 (IQR: 17.1, 19.9), respectively. Compared to the lower-RDW group, patients with higher RDW typically had a higher BMI, and greater occurrence of CHF, diabetes, chronic pulmonary disease, renal disease, mild liver disease, and metastatic solid tumors. They also exhibited lower levels of WBC, RBC, Hb, HCT, serum sodium, GLU, MCH, MCHC, and MCV. With increasing RDW index, hospital stay length (3.75 days vs. 3.67 days vs. 4.29 days vs. 4.92 days) increased gradually, as well as the 6-month death (5.8% vs. 8.0% vs. 15.5% vs. 26.5%; *P* < 0.001), 1-year death (9.7% vs. 13.0% vs. 25.0% vs. 37.8%; *P* < 0.001), and 3-year death (14.4% vs. 17.5% vs. 15.5% vs. 44.8%; *P* < 0.001) rates.

Differences in baseline characteristics between individuals who survived and those who did not survive their hospital stays are shown in Table 2. Individuals in the non-survival group had older average ages, a lower rate of breast surgery, and a greater occurrence of CHF, chronic pulmonary disease, mild liver disease, renal disease, and metastatic solid tumors (*P* < 0.05). As for laboratory parameters, the non-surviving group exhibited lower levels of Hb, chloride, serum sodium, GLU, and MCHC and higher levels of AG and BUN (*P* < 0.05). There were no discernible changes in BMI, bicarbonate levels, RBC count, HCT, or MCH. Compared to the survival group, the non-survival group’s RDW Index was substantially higher (15.8 vs. 14.0%, *P* < 0.001). The distribution of the RDW level, stratified by mortality status, for fatalities that occurred within six months, one year, and three years is shown in S1 Fig.

**Table 2:** Baseline characteristics of the survivors and non-survivors groups.

Categories	Overall (N = 939)	Survivors (N = 539)	Non-Survivors (N = 350)	*p*-value
Age, years	64.95 (14.05)	64.18 (13.89)	68.28 (13.46)	<0.001
BMI, kg/m^2^	27.94 (23.83, 33.25)	28.19 (23.86,33.66)	27.60 (23.78, 33.14)	0.510
White, n (%)	657 (70.0)	403 (68.4)	254 (72.6)	0.180
Radiation, n (%)	37 (3.9)	14 (2.4)	23 (6.6)	0.001
Medication, n (%)	13 (1.4)	8 (1.4)	5 (1.4)	0.929
Breast Surgery, n (%)	131 (14)	117 (19.9)	14 (4.0)	<0.001
Comorbidities, n (%)				
Myocardial infarction	44 (4.7)	25 (4.2)	19 (5.4)	0.406
Congestive heart failure	109 (11.6)	45 (7.6)	64 (18.3)	<0.001
Peripheral vascular disease	28 (3.0)	13 (2.2)	15 (4.3)	0.070
Cerebrovascular disease	50 (5.3)	28 (4.8)	22 (6.3)	0.312
Chronic pulmonary disease	170 (18.1)	89 (15.1)	81 (23.1)	0.002
Rheumatic disease	26 (2.8)	14 (2.4)	12 (3.4)	0.342
Mild liver disease	59 (6.3)	32 (5.4)	27 (7.7)	0.164
Diabetes without CC	147 (15.7)	91 (15.4)	56 (16.0)	0.823
Diabetes with CC	53 (5.6)	29 (4.9)	24 (6.9)	0.214
Renal disease	95 (10.1)	49 (8.3)	46 (13.1)	0.018
Severe liver disease	28 (3.0)	11 (1.9)	17 (4.9)	0.009
Metastatic solid tumor	399 (42.5)	164 (27.8)	235 (67.1)	<0.001
Laboratory tests				
AG, mmol/L	13.73 (12.00, 15.00)	13.73 (12.00, 15.00)	14.00 (12.00, 16.00)	<0.001
Bicarbonate, mmol/L	25.01 (23.00,27.00)	25.01 (24.00, 27.00)	25.00 (23.00, 27.00)	0.791
WBC, ×10^9^/L	7.4 (4.9, 10.8)	7.2 (4.7, 10.7)	6.9 (4.8, 10.6)	0.319
RBC, ×10^12^/L	3.46 (3.05, 3.92)	3.48 (3.08, 3.94)	3.37 (2.92, 3.92)	0.074
PLT, ×10^9^/L	218 (162, 285)	220 (165, 289)	219 (149, 302)	0.740
Hb, g/dL	10.3 (9.0, 11.6)	10.5 (9.1, 11.7)	9.9 (8.7, 11.4)	0.004
HCT, %	31.4 (27.7, 35.4)	31.8 (27.9, 35.7)	30.5 (27, 35)	0.056
Chloride, mmol/L	103.0 (100.0, 105.0)	103.0 (102.0, 106.0)	102.4 (99.0, 105.0)	<0.001
Calcium, mmol/L	8.68 (8.30, 9.00)	8.68 (8.40, 9.00)	8.68 (8.20, 9.03)	0.743
Potassium, mmol/L	4.01 (3.70, 4.20)	4.01 (3.70, 4.20)	4.00 (3.70, 4.33)	0.790
Sodium, mmol/L	138.6 (137.0, 141.0)	138.6 (137.0, 141.0)	138.0 (136.0, 141.0)	<0.001
GLU, mg/dL	115.0 (95.0, 131.0)	120.0 (98.0, 129.0)	107.0 (91.0, 135.3)	0.004
Creatinine, mg/dL	0.80 (0.60, 0.95)	0.80 (0.60, 0.95)	0.70 (0.60, 1.10)	0.288
BUN, mg/dL	15.00 (10.00, 18.00)	14.00 (9.50, 17.23)	15.00 (11.00, 23.00)	<0.001
MCH, pg	30.0 (28.2, 31.3)	30.0 (28.5 31.4)	29.6 (27.9, 31.3)	0.060
MCHC, %	32.9 (31.8, 33.7)	33.0 (32.0, 34.0)	32.6 (31.4, 33.6)	<0.001
MCV, fL	91 (87, 95)	91 (86, 95)	91 (87, 95)	0.752
RDW index, %	14.6 (13.4, 16.4)	14.0 (13.1, 15.4)	15.8 (14.4, 17.3)	<0.001

Abbreviation: BMI, body mass index; CC; complications or comorbidities; AG, anion gap; WBC, white blood cell; RBC, red blood cell; PLT, platelet; Hb, hemoglobin; HCT, hematocrit; GLU, glucose; BUN, blood urea nitrogen; MCH, mean corpuscular hemoglobin; MCHC, mean corpuscular hemoglobin concentration; MCV, mean corpuscular volume; RDW, red blood cell distribution width.

### Primary outcomes

**Fig 2:**
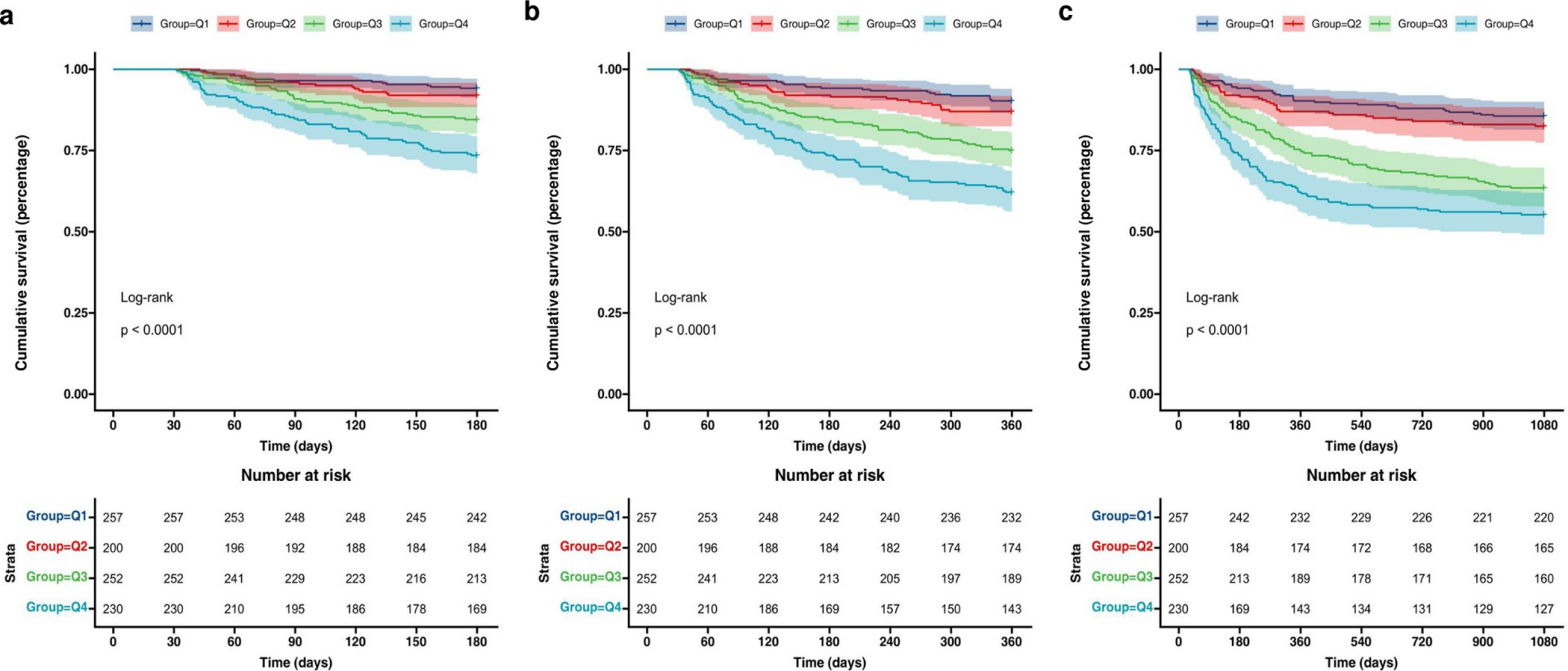
Kaplan–Meier survival analysis curves for all-cause mortality. Footnote RDW index quartiles: Q1 (10.8–13.4), Q2 (13.4–14.6), Q3 (14.6–16.4), Q4 (16.4–26.8). Kaplan–Meier curves showing a cumulative probability of all-cause mortality according to groups at 6 months (a), 1 year (b), and 3 years (c).

The Kaplan-Meier survival analysis curves for the incidence of the primary outcome in each quartile of RDW are shown in Fig 2. In general, patients with elevated RDW have an increased risk of in-hospital death. At 6-month, 1-year, and 3-year extended follow-ups, individuals with a high RDW index exhibited significantly higher overall mortality rates than those with a low RDW index (all log-rank *P* < 0.001; Fig 2).

ROC analysis was performed to evaluate the clinical predictive value of the RDW index for in-hospital mortality. However, we observed that the effectiveness of RDW in predicting all-cause mortality was suboptimal (AUC for 6-month death: 0.704, *P* < 0.001; AUC for 1-year death: 0.691, *P* < 0.001; and AUC for 3-year death: 0.679, *P* < 0.001). The cutoff values for RDW were 15.75, 15.35, and 14.55, respectively (S2 Fig).

**Fig 3:**
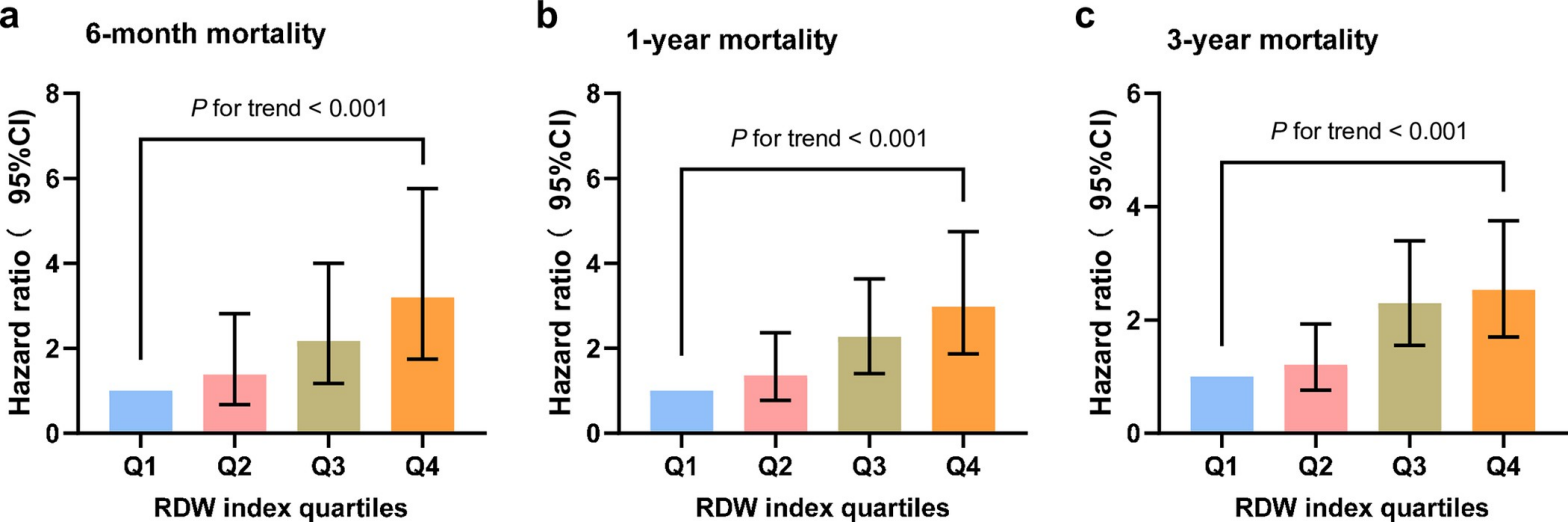
Hazard ratios (95% CIs) for hospital mortality according to RDW index quartiles of model 3. Hazard ratios (95% CIs) for 6-month (a), 1-year (b), and 3-year (c) mortality according to RDW index quartiles after adjusting for age, race, BMI, radiation, medication, breast surgery, MI, CHF, peripheral vascular disease, cerebrovascular disease, chronic pulmonary disease, rheumatic disease, mild liver disease, diabetes without cc, diabetes with cc, renal disease, severe liver disease, metastatic solid tumor. Error bars indicate 95% CIs. The first quartile is the reference. CI, confidence interval. Abbreviations: RDW, red cell distribution width.

Cox proportional hazards analysis was performed to examine the relationship between RDW and overall mortality. It was demonstrated that when RDW was taken as a continuous variable, in the unadjusted model (HR, 1.226 [95% CI 1.166–1.289]; *P* < 0.001), partially adjusted model (HR, 1.251 [95% CI 1.187–1.319]; *P* < 0.001), and fully adjusted model (HR, 1.187 [95% CI 1.114–1.264]; *P* < 0.001), RDW was significantly correlated with 6-month, 1-year, and 3-year mortality. When RDW was considered as a nominal variable, in the three established models, patients within higher RDW quartiles were at a considerably elevated risk of 6-month death: unadjusted model (HR, 5.136 [95% CI 2.919–9.037]; *P* <0.001), partially adjusted model (HR, 5.321[95% CI 3.011–9.403]; *P* <0.001), and completely adjusted model (HR, 3.197 [95% CI 1.745–5.762]; *P* <0.001), compared to participants in the bottom quartile of RDW; and showed a tendency to rise with the RDW index (Table 3, Fig 3A). Similar outcomes were found in the multivariate Cox analyses conducted to evaluate the association between the RDW index and the 1-year and 3-year mortality rates (Table 3, Fig 3B and 3C).

**Table 3:** Cox proportional hazard ratios (HR) for all-cause mortality.

Categories	Model 1			Model 2			Model 3		
	HR (95% CI)	*p*-value	*p* for trend	HR (95% CI)	*p*-value	*p* for trend	HR (95% CI)	*p*-value	*p* for trend
**6-month mortality**									
Continuous variable per 1 unit	1.226 (1.166, 1.289)	<0.001		1.251 (1.187, 1.319)	<0.001		1.187 (1.114, 1.264)	<0.001	
Quartile			<0.001			<0.001			<0.001
Q1 (N = 257)	Ref.			Ref.			Ref.		
Q2 (N = 200)	1.388 (0.686, 2.807)	0.362		1.373 (0.677, 2.785)	0.379		1.376 (0.673, 2.816)	0.382	
Q3 (N = 252)	2.792 (1.539, 5.064)	<0.001		2.741 (1.505, 4.991)	<0.001		2.168 (1.175, 4.001)	0.013	
Q4 (N = 230)	5.136 (2.919, 9.037)	<0.001		5.321 (3.011, 9.403)	<0.001		3.197 (1.745, 5.762)	<0.001	
**1-year mortality**									
Continuous variable per 1 unit	1.200 (1.150, 1.251)	<0.001		1.225 (1.173, 1.280)	<0.001		1.158 (1.099, 1.219)	<0.001	
Quartile			<0.001			<0.001			<0.001
Q1 (N = 257)	Ref.			Ref.			Ref.		
Q2 (N = 200)	1.365 (0.788, 2.364)	0.267		1.380 (0.795, 2.394)	0.252		1.357 (0.778, 2.368)	0.283	
Q3 (N = 252)	2.804 (1.764, 4.457)	<0.001		2.819 (1.768, 4.494)	<0.001		2.263 (1.408, 3.636)	<0.001	
Q4 (N = 230)	4.719 (3.024, 7.364)	<0.001		5.037 (3.218, 7.884)	<0.001		2.978 (1.867, 4.748)	<0.001	
**3-year mortality**									
Continuous variable per 1 unit	1.177 (1.134, 1.222)	<0.001		1.199 (1.153, 1.246)	<0.001		1.127 (1.077, 1.180)	<0.001	
Quartile			<0.001			<0.001			<0.001
Q1 (N = 257)	Ref.			Ref.			Ref.		
Q2 (N = 200)	1.248 (0.786, 1.980)	0.348		1.225 (0.770, 1.949)	0.392		1.208 (0.757, 1.928)	0.429	
Q3 (N = 252)	2.880 (1.966, 4.218)	<0.001		2.804 (1.908, 4.122)	<0.001		2.295 (1.551, 3.396)	<0.001	
Q4 (N = 230)	3.999 (2.746, 5.825)	<0.001		4.156 (2.845, 6.071)	<0.001		2.526 (1.701, 3.750)	<0.001	

RDW index: Q1 (10.8–13.4), Q2 (13.4–14.6), Q3 (14.6–16.4), Q4 (16.4–26.8)

Model 1: unadjusted

Model 2: adjusted for age, race, BMI

Model 3: adjusted for age, race, BMI, radiation, medication, breast surgery, MI, CHF, PVD, CVD, chronic pulmonary disease, rheumatic disease, mild liver disease, diabetes without cc, diabetes with cc, renal disease, severe liver disease, metastatic solid tumor.

Abbreviations: HR, hazard ratio; CI, confidence interval.

**Fig 4:**
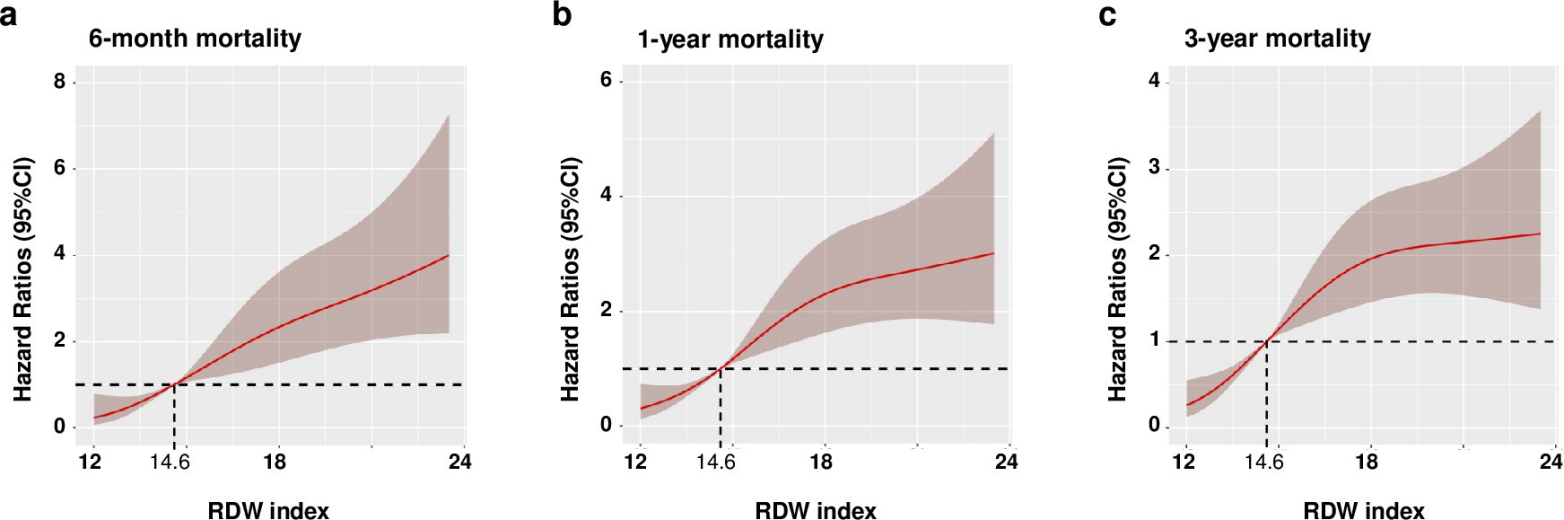
Restricted cubic spline curves for the RDW hazard ratio. Heavy central lines represent the estimated adjusted hazard ratios, with shaded ribbons denoting 95% confidence intervals. RDW index 14.6 was selected as the reference level represented by the vertical dotted lines. The horizontal dotted lines represent the hazard ratio of 1.0. (a) Restricted cubic spline for 6-month mortality. (b) Restricted cubic spline for 1-year mortality. (c) Restricted cubic spline for 3-year mortality. HR, hazard ratio; CI, confidence interval. Abbreviations: RDW, red cell distribution width.

Furthermore, the application of RCS regression models helped ascertain a significant association, indicating that elevated RDW levels (> 14.6) were linked to an increased likelihood of mortality (Fig 4).

### Subgroup analysis

Considering the various subgroups of the enrolled patients that might be potentially influencing factors, we investigated how the RDW level was risk-stratified for the main outcomes meticulously, taking into account various subgroups including age, BMI, race, CHF, chronic pulmonary disease, and renal disease. (Table 4). The RDW index was significantly associated with an increased risk of 6-month mortality in specific subgroups of patients with breast cancer. These subgroups included individuals of non-white (HR, 4.599; 95% CI 1.043–20.286), individuals aged ≥65 years (HR 3.479; 95% CI 1.607–7.532), individuals with BMI ≥30 kg/m^2^ (HR 2.441; 95% CI 0.082–7.433), individuals without CHF (HR 3.866; 95% CI 1.975–7.569), and individuals without renal disease (HR 3.631; 95% CI 1.925–6.850) (all *P* < 0.05). Similar associations were observed in the stratified analyses of the RDW index and the 1-year, and 3-year mortality rates. However, there was no significant difference in the cross-stratification of RDW quartiles by age, BMI, race, chronic pulmonary disease, or renal disease (*P* for interaction > 0.05), suggesting that our subgroup analysis was relatively stable and less affected by confounding factors. Interestingly, the predictive ability of RDW appeared to be even more remarkable in patients without CHF than those with CHF (HR 3.866; [95% CI 1.975–7.569] vs. HR 1.410; [95% CI 0.325–6.121], *P* for interaction = 0.026).

**Table 4:** Multivariate Cox analyses for categorized RDW and clinical outcomes in BC patients in different subgroups according to the fully adjusted model (Model 3).

Subgroups		Results [HR/OR, (95%CI), *p*-value]					
		6-month mortality	*p*-value for interaction	1-year mortality	*p*-value for interaction	3-year mortality	*p*-value for interaction
Age≥65	Q1	Ref. 0.004	0.775	Ref. <0.001	0.486	Ref. <0.001	0.094
	Q2	1.413 (0.579, 3.450), 0.448		1.357 (0.690, 2.670), 0.377		0.938 (0.527, 1.671), 0.829	
	Q3	2.444 (1.125, 5.309), 0.024		2.138 (1.171, 3.903), 0.013		1.820 (1.121, 2.953), 0.015	
	Q4	3.479 (1.607, 7.532), 0.002		3.159 (1.744, 5.725), <0.001		2.292 (1.399, 3.756), <0.001	
Age<65	Q1	Ref. 0.172		Ref. 0.047		Ref. 0.007	
	Q2	1.325 (0.378, 4.647), 0.660		1.307 (0.471, 3.631), 0.607		2.024 (0.883, 4.639), 0.096	
	Q3	1.563 (0.545, 4.488), 0.406		2.227 (0.989, 5.017), 0.053		3.096 (1.523, 6.295), 0.002	
	Q4	2.549 (0.946, 6.868), 0.064		2.753 (1.247, 6.079), 0.012		3.228 (1.592, 6.544), 0.001	
BMI<30 kg/m^2^	Q1	Ref. <0.001	0.956	Ref. <0.001	0.590	Ref. <0.001	0.472
	Q2	1.234 (0.513, 2.969), 0.639		1.231 (0.642, 2.358), 0.532		1.387 (0786, 2.415), 0.262	
	Q3	2.452 (1.194, 5.037), 0.015		2.107 (1.213, 3.655), 0.008		2.190 (1.344, 3.569), 0.002	
	Q4	3.680 (1.800, 7.525), <0.001		2.862 (1.657, 4.944), <0.001		2.861 (1.759, 4.656), <0.001	
BMI≥30 kg/m^2^	Q1	Ref. 0.187		Ref. 0.022		Ref. 0.013	
	Q2	1.077 (0.293, 3.959), 0.911		1.335 (0.425, 4.192), 0.621		0.817 (0.341, 1.959), 0.651	
	Q3	1.387 (0.436, 4.414), 0.580		2.398 (0.885, 6.494), 0.085		2.161 (1.084, 4.309), 0.029	
	Q4	2.441 (0.802, 7.433), 0.116		3.473 (1.320, 9.137), 0.012		2.056 (1.027, 4.115), 0.042	
Race (white)	Q1	Ref. 0.002	0.222	Ref. <0.001	0.564	Ref. <0.001	0.441
	Q2	1.121 (0.482, 2.609), 0.791		1.176 (0.620, 2.232), 0.620		1.127 (0.657, 1.935), 0.664	
	Q3	2.456 (1.260, 4.786), 0.008		2.404 (1.423, 4.062), 0.001		2.475 (1.594, 3.845), <0.001	
	Q4	2.964 (1.507, 5.828),0.002		2.825 (1.664, 4.796), <0.001		2.384 (1.509, 3.765), <0.001	
Race (other)	Q1	Ref. 0.037		Ref. 0.042		Ref. 0.017	
	Q2	2.150 (0.424, 10.888), 0.355		1.806 (0.531, 6.148), 0.344		1.257 (0.472, 3.349), 0.647	
	Q3	1.725 (0.349, 8.522), 0.504		2.227 (0.718, 6.911), 0.166		1.940 (0.816, 4.612), 0.134	
	Q4	4.599 (1.043, 20.286), 0.044		3.776 (1.290, 11.055), 0.015		3.034 (1.311, 7.023), 0.010	
Congestive heart failure (+)	Q1	Ref. 0.473	0.026	Ref. 0.486	0.022	Ref. 0.978	0.016
	Q2	1.733 (0.402, 7.482), 0.461		2.105 (0.538, 8.239), 0.285		1.018 (0.347, 2.990), 0.974	
	Q3	0.677 (0.156, 2.945), 0.603		1.202 (0.309, 4.670), 0.791		0.894 (0.315, 2.539), 0.833	
	Q4	1.410 (0.325, 6.121), 0.646		2.101 (0.528, 8.363), 0.292		1.052 (0.349, 3.170), 0.929	
Congestive heart failure (-)	Q1	Ref. <0.001		Ref. <0.001		Ref. <0.001	
	Q2	1.037 (0.431, 2.494), 0.935		1.054 (0.555, 2.002), 0.871		1.070 (0.623, 1.836), 0.807	
	Q3	2.811 (1.416, 5.580), 0.003		2.559 (1.536, 4.261), <0.001		2.752 (1.791, 4.228), <0.001	
	Q4	3.866 (1.975, 7.569), <0.001		3.232 (1.955, 5.342), <0.001		3.009 (1.954, 4.635), <0.001	
Chronic pulmonary disease (+)	Q1	Ref. 0.158	0.327	Ref. 0.058	0.679	Ref. 0.030	0.730
	Q2	1.170 (0.208, 76.592), 0.859		1.118 (0.290, 4.300), 0.871		0.932 (0.289, 3.005), 0.906	
	Q3	2.087 (0.541, 8.055), 0.286		1.868 (0.674, 5.171), 0.229		2.229 (0.948, 5.241), 0.066	
	Q4	3.456 (0.908, 13.156), 0.069		3.160 (1.178, 8.477), 0.022		2.841 (1.206, 6.694), 0.017	
Chronic pulmonary disease (-)	Q1	Ref. 0.002		Ref. <0.001		Ref. <0.001	
	Q2	1.374 (0.610, 3.095), 0.443		4.139 (0.510, 33.621), 0.184		1.314 (0.781, 2.210), 0.304	
	Q3	2.155 (1.054, 4.409), 0.035		0.758 (0.105, 5.460), 0.783		2.360 (1.500, 3.712), <0.001	
	Q4	3.283 (1.639, 6.576), <0.001		0.642 (0.077, 5.375), 0.682		2.661 (1.687, 4.198), <0.001	
Renal disease (+)	Q1	Ref. 0.567	0.135	Ref. 0.187	0.084	Ref. 0.780	0.239
	Q2	0.685 (0.026, 17.783), 0.820		1.374 (0.610, 3.095), 0.443		1.266 (0.352, 4.561), 0.718	
	Q3	0.254 (0.019, 3.463), 0.304		2.155 (1.054, 4.409), 0.035		0.737 (0.249, 2.183), 0.582	
	Q4	0.139 (0.007, 2.644), 0.189		3.283 (1.639, 6.576), <0.001		0.676 (0.177, 2.584), 0.567	
Renal disease (-)	Q1	Ref. <0.001		Ref. <0.001		Ref. <0.001	
	Q2	1.478 (0.697, 3.136), 0.309		1.236 (0.685, 2.229), 0.482		1.309 (0.786, 2.180), 0.301	
	Q3	2.542 (1.324, 4.880), 0.005		2.501 (1.529, 4.093), <0.001		2.789 (1.818, 4.278), <0.001	
	Q4	3.631 (1.925, 6.850), <0.001		3.139 (1.934, 5.096), <0.001		3.042 (1.983, 4.668), <0.001	

RDW index: Q1 (10.8–13.4), Q2 (13.4–14.6), Q3 (14.6–16.4), Q4 (16.4–26.8)

Model 3: adjusted for age, race, BMI, radiation, medication, breast surgery, MI, CHF, PVD, CVD, chronic pulmonary disease, rheumatic disease, mild liver disease, diabetes without cc, diabetes with cc, renal disease, severe liver disease, metastatic solid tumor.

Abbreviations: HR, hazard ratio; OR, odds ratio; CI, confidence interval; BMI, body mass index.

## Discussion

Our study suggests that increased RDW is a robust and independent predictor of higher mortality in patients with breast cancer. The formidable association between elevated RDW and all-cause mortality remains prominent even after adjusting for potential interfering factors. Owing to its availability and cost-effectiveness in routine blood examinations, our investigation proposes that RDW could serve as a novel, reliable indicator in clinics, helping to identify patients with breast cancer at risk of unfavorable prognosis.

RDW is a common measurement of RBC included in the complete blood count (CBC), reflecting the heterogeneity of the circulating red blood cell volume. The abnormal elevation in RDW suggests that inflammatory cytokines stimulate the premature release of immature large red blood cells into the peripheral blood circulation [18], leading to an increase in red blood cell volume variation. Clinical studies have shown that, compared with healthy subjects, there were significant differences in RDW values between patients treated for cancers and those with non-cancer diseases such as hematologic [19], cardiovascular [10], and systemic diseases. Changes in RDW are particularly evident in cardiovascular diseases, and its elevation has been demonstrated as a reliable indicator of negative consequences in a variety of cerebrovascular diseases, including heart failure, pulmonary embolism, ischemic stroke, hemorrhagic stroke, and coronary heart disease among others [20].

Cancer-related chronic inflammation is a key feature of tumor development. RDW has emerged as a reliable marker for systemic inflammatory response in various malignancies, consistently linked to adverse outcomes in extensive research. In 2009, a community-based prospective study reported a strong and independent association between higher RDW and the risk of death from cancer [21]. A meta-analysis by Wang et al. identified a negative correlation between a pre-treatment RDW threshold of 13%-14% and poor survival outcomes [22], while Ines et al. associated high RDW with adverse prognostic factors in patients with Hodgkin’s lymphoma (HL) [23]. Warwick et al. examined the data of 917 patients who underwent surgery for non-small-cell lung carcinoma and confirmed that a preoperative RDW-CV of >15.3% was a significant risk factor for postoperative death (*P* = 0.001) and survival (*P* = 0.0001) [24]. In colorectal cancer, a high RDW level (≥13.5%) was reported as an additional separate indicator of both cause-specific survival (CSS) and OS [25], with a significant reduction in 10-year OS among patients with high RDW [26]. These studies all suggest that RDW is an important biomarker for cancer. However, the exact biological mechanism underlying the association between RDW and all-cause mortality risk in patients with cancer remains unclear. One hypothesis is that increased oxidative stress may reduce red blood cell survival and increase immature red blood cells in circulation, leading to increased RDW [27]. Another plausible explanation is that elevated RDW levels in cancer patients may be attributed to prolonged inflammatory responses and increased circulating cytokine levels, possibly causing damage to red blood cell membranes and influencing erythropoietin production, ultimately leading to an increase in RDW [28]. Additionally, due to the limited specificity of RDW in cancer diagnosis, its application has certain constraints. Therefore, scholars have mainly focused on investigating the value of RDW in the prognosis assessment of cancer patients.

Researchers have disagreed on the predictive value of elevated RDW for breast cancer. Previous studies have shown that high RDW levels can be observed in patients with breast cancer [29], especially in postmenopausal women [30], compared to healthy individuals [31]. Therefore, RDW levels can effectively distinguish patients with breast cancer from healthy individuals [32]. Furthermore, RDW levels were significantly higher in patients with breast cancer than in patients with benign breast fibroadenomas [33]. Seretis et al. observed elevated preoperative RDW in breast cancer patients compared to those with breast fibroadenoma. The heightened RDW correlated with tumor size, metastatic lymph nodes, and HER2 overexpression, suggesting its potential in distinguishing benign from malignant breast tumors [12]. Some scholars believe that RDW might act as a biomarker for evaluating the metastatic capability of tumors [34], and an escalated RDW before treatment was found to serve as a standalone variable that negatively affected the survival rate of young females with breast cancer [14]. Moreover, Yao et al. showed that high pretreatment RDW levels in patients with breast cancer were associated with poorer OS and DFS [35], suggesting that RDW may be a potential predictor of poor prognosis in all patients. Yoo et al. [15] further investigated preoperative hematologic indicators in patients with breast cancer and found that a markedly elevated RDW >13.5% was the most robust predictor of postoperative mortality. Surpassing this RDW threshold was associated with about a 1.7-fold increase in both recurrence likelihood and death risk. In contrast, Zou et al. showed no difference in RDW values between patients with breast cancer and patients with breast fibroadenoma, but RDW levels were significantly negatively associated with the histological grade of breast cancer [36]. Another retrospective study showed that pre-treatment RDW values in patients with breast cancer were not significantly associated with survival, whereas increased RDW and levels after surgery and adjuvant therapy were associated with poor DFS and OS [37, 38]. Fu et al. [39] demonstrated that elevated RDW was significantly associated with poor prognosis in triple-negative breast cancer, but not in the Luminal A subtype. Although the conclusions from these studies vary, we should be aware of the value of RDW markers in adjuvant diagnosis, differentiation between benign and malignant breast cancer, and prognostic judgment.

### Strengths and limitations

Our study observed a significant increase in short-term, medium-term, and long-term in-hospital mortality rates among patients with breast cancer exhibiting elevated RDW, aligning with findings from some previous studies. In contrast to prior studies, our study stands out in the exploration of the relationship between RDW and in-hospital mortality in patients with breast cancer through several distinctive features. Firstly, by incorporating data from a large sample of patients with breast cancer over the last 11 years into the MIMIC-IV database, we used multivariate covariate analysis to account for distinct baseline characteristics linked to RDW, intending to eliminate the impact of confounding variables (such as age, race, BMI, medication, surgery, and comorbidities). Employing advanced statistical methods and meticulous adjustment for confounding factors enhances the robustness and reliability of our findings. The persistence of the significant association between RDW and in-hospital mortality, even after accounting for these confounding risk factors, underscores the utility of RDW as a precise tool for risk assessment in clinical practice. Secondly, our comprehensive subgroup analyses provide a nuanced understanding of the specific associations between RDW and in-hospital mortality in various subpopulations, offering valuable insights for personalized treatment decision-making. Additionally, our study extends beyond the conventional focus on younger patients or specific phases of breast cancer treatment, integrating RDW into the broader clinical context over an extended period. This approach enables a more comprehensive analysis of RDW’s potential impact on patient care, risk stratification, and clinical decision-making. In conclusion, our study not only confirms and refines the association between RDW and in-hospital mortality in breast cancer patients but also contributes methodological nuances, broadening the understanding of RDW’s significance through meticulous adjustment, subgroup analyses, and comprehensive clinical contextualization. The methodological rigor and comprehensive exploration distinguish our study, enhancing its applicability and contributing meaningfully to the scientific discourse in this field. The study has several limitations. Firstly, the MIMIC database lacks staging information for patients with breast cancer, preventing the incorporation of this crucial factor into our analysis. The tumor stage is pivotal in understanding the extent of cancer progression and tailoring appropriate treatment strategies. To mitigate this limitation, future research endeavors could involve collaboration with multiple medical centers or databases that encompass comprehensive patient data, including detailed staging information. Secondly, due to the limited availability of postoperative RDW records, with only 11 samples, the clear distinction between preoperative and postoperative RDW data was challenging. Recognizing postoperative RDW as a dynamic indicator reflecting the interplay between systemic inflammatory and immune responses after surgery adds complexity to its interpretation and differentiation from preoperative RDW. Additionally, the reliance on data from a single center introduces limitations in terms of generalizability. To address these constraints and enhance the robustness of our findings, a future multicenter or prospective study with a larger sample size is essential.

## Conclusion

In conclusion, our study suggests that RDW, as a simple, inexpensive, and readily available routine blood test, may serve as a significant risk predictor of all-cause mortality in patients with breast cancer. By understanding the relationship between RDW and the survival outcome, we can comprehensively assess the overall health status of breast cancer patients. This knowledge allows for strengthened monitoring and management of high-risk patients in future clinical practice.

## Supporting information

S1 Fig The distribution of the RDW index stratified by mortality status. Abbreviations: RDW, red cell distribution width.
(TIF)

S2 Fig Receiver Operating Characteristic curves for all-cause mortality. Abbreviations: AUC, area under curve.
(TIF)

S1 Table Missing number for risk variables and outcome variables. (DOCX)

S1 Raw data (CSV)

## References

[1] Siegel RL, Giaquinto AN, Jemal A. Cancer statistics, 2024. CA Cancer J Clin. 2024;74(1):12–49. doi: 10.3322/caac.21820 . 38230766

[2] Giaquinto AN, Sung H, Miller KD, Kramer JL, Newman LA, Minihan A, et al. Breast Cancer Statistics, 2022. CA Cancer J Clin. 2022;72(6):524–41. doi: 10.3322/caac.21754 . 36190501

[3] Lukasiewicz S, Czeczelewski M, Forma A, Baj J, Sitarz R, Stanislawek A. Breast Cancer-Epidemiology, Risk Factors, Classification, Prognostic Markers, and Current Treatment Strategies-An Updated Review. Cancers (Basel). 2021;13(17):4287. doi: 10.3390/cancers13174287 . PubMed Central PMCID: PMC8428369. 34503097 PMC8428369

[4] Kohler BA, Sherman RL, Howlader N, Jemal A, Ryerson AB, Henry KA, et al. Annual Report to the Nation on the Status of Cancer, 1975–2011, Featuring Incidence of Breast Cancer Subtypes by Race/Ethnicity, Poverty, and State. J Natl Cancer Inst. 2015;107(6):djv048. doi: 10.1093/jnci/djv048 . PubMed Central PMCID: PMC4603551. 25825511 PMC4603551

[5] Li Y, Yang D, Yin X, Zhang X, Huang J, Wu Y, et al. Clinicopathological Characteristics and Breast Cancer-Specific Survival of Patients With Single Hormone Receptor-Positive Breast Cancer. JAMA Netw Open. 2020;3(1):e1918160. doi: 10.1001/jamanetworkopen.2019.18160 . PubMed Central PMCID: PMC6991239. 31899528 PMC6991239

[6] Obr AE, Edwards DP. The biology of progesterone receptor in the normal mammary gland and in breast cancer. Mol Cell Endocrinol. 2012;357(1–2):4–17. doi: 10.1016/j.mce.2011.10.030 . PubMed Central PMCID: PMC3318965. 22193050 PMC3318965

[7] Nishimura R, Osako T, Okumura Y, Hayashi M, Toyozumi Y, Arima N. Ki-67 as a prognostic marker according to breast cancer subtype and a predictor of recurrence time in primary breast cancer. Exp Ther Med. 2010;1(5):747–54. doi: 10.3892/etm.2010.133 . PubMed Central PMCID: PMC3445951. 22993598 PMC3445951

[8] Salvagno GL, Sanchis-Gomar F, Picanza A, Lippi G. Red blood cell distribution width: A simple parameter with multiple clinical applications. Crit Rev Clin Lab Sci. 2015;52(2):86–105. doi: 10.3109/10408363.2014.992064 . 25535770

[9] Kim M, Lee CJ, Kang HJ, Son NH, Bae S, Seo J, et al. Red cell distribution width as a prognosticator in patients with heart failure. ESC Heart Fail. 2023;10(2):834–45. doi: 10.1002/ehf2.14231 . PubMed Central PMCID: PMC10053156. 36460487 PMC10053156

[10] Li N, Zhou H, Tang Q. Red Blood Cell Distribution Width: A Novel Predictive Indicator for Cardiovascular and Cerebrovascular Diseases. Dis Markers. 2017;2017:7089493. doi: 10.1155/2017/7089493 . PubMed Central PMCID: PMC5606102. 29038615 PMC5606102

[11] Kim KM, Nerlekar R, Tranah GJ, Browner WS, Cummings SR. Higher red cell distribution width and poorer hospitalization-related outcomes in elderly patients. J Am Geriatr Soc. 2022;70(8):2354–62. doi: 10.1111/jgs.17819 . 35506925

[12] Seretis C, Seretis F, Lagoudianakis E, Gemenetzis G, Salemis NS. Is red cell distribution width a novel biomarker of breast cancer activity? Data from a pilot study. J Clin Med Res. 2013;5(2):121–6. doi: 10.4021/jocmr1214w . PubMed Central PMCID: PMC3601498. 23518817 PMC3601498

[13] Takeuchi H, Abe M, Takumi Y, Hashimoto T, Miyawaki M, Okamoto T, et al. Elevated red cell distribution width to platelet count ratio predicts poor prognosis in patients with breast cancer. Sci Rep. 2019;9(1):3033. doi: 10.1038/s41598-019-40024-8 . PubMed Central PMCID: PMC6395769. 30816333 PMC6395769

[14] Huang DP, Ma RM, Xiang YQ. Utility of Red Cell Distribution Width as a Prognostic Factor in Young Breast Cancer Patients. Medicine (Baltimore). 2016;95(17):e3430. doi: 10.1097/MD.0000000000003430 . PubMed Central PMCID: PMC4998693. 27124030 PMC4998693

[15] Yoo YC, Park S, Kim HJ, Jung HE, Kim JY, Kim MH. Preoperative Routine Laboratory Markers for Predicting Postoperative Recurrence and Death in Patients with Breast Cancer. J Clin Med. 2021;10(12):2610. doi: 10.3390/jcm10122610 . PubMed Central PMCID: PMC8231951. 34199276 PMC8231951

[16] Johnson AEW, Bulgarelli L, Shen L, Gayles A, Shammout A, Horng S, et al. MIMIC-IV, a freely accessible electronic health record dataset. Sci Data. 2023;10(1):1. doi: 10.1038/s41597-022-01899-x . PubMed Central PMCID: PMC9810617. 36596836 PMC9810617

[17] Evans TC, Jehle D. The red blood cell distribution width. J Emerg Med. 1991;9 Suppl 1:71–4. doi: 10.1016/0736-4679(91)90592-4 . 1955687

[18] Lippi G, Filippozzi L, Montagnana M, Salvagno GL, Franchini M, Guidi GC, et al. Clinical usefulness of measuring red blood cell distribution width on admission in patients with acute coronary syndromes. Clin Chem Lab Med. 2009;47(3):353–7. doi: 10.1515/cclm.2009.066 . 19676148

[19] Ai L, Mu S, Hu Y. Prognostic role of RDW in hematological malignancies: a systematic review and meta-analysis. Cancer Cell Int. 2018;18(1):61. doi: 10.1186/s12935-018-0558-3 . PubMed Central PMCID: PMC5914059. 29713244 PMC5914059

[20] Parizadeh SM, Jafarzadeh-Esfehani R, Bahreyni A, Ghandehari M, Shafiee M, Rahmani F, et al. The diagnostic and prognostic value of red cell distribution width in cardiovascular disease; current status and prospective. Biofactors. 2019;45(4):507–16. doi: 10.1002/biof.1518 . 31145514

[21] Perlstein TS, Weuve J, Pfeffer MA, Beckman JA. Red blood cell distribution width and mortality risk in a community-based prospective cohort. Arch Intern Med. 2009;169(6):588–94. doi: 10.1001/archinternmed.2009.55 . PubMed Central PMCID: PMC3387573. 19307522 PMC3387573

[22] Wang PF, Song SY, Guo H, Wang TJ, Liu N, Yan CX. Prognostic role of pretreatment red blood cell distribution width in patients with cancer: A meta-analysis of 49 studies. J Cancer. 2019;10(18):4305–17. doi: 10.7150/jca.31598 . PubMed Central PMCID: PMC6691718. 31413750 PMC6691718

[23] Herraez I, Bento L, Del Campo R, Sas A, Ramos R, Ibarra J, et al. Prognostic Role of the Red Blood Cell Distribution Width (RDW) in Hodgkin Lymphoma. Cancers (Basel). 2020;12(11):3262. doi: 10.3390/cancers12113262 . PubMed Central PMCID: PMC7694294. 33158258 PMC7694294

[24] Warwick R, Mediratta N, Shackcloth M, Shaw M, McShane J, Poullis M. Preoperative red cell distribution width in patients undergoing pulmonary resections for non-small-cell lung cancer. Eur J Cardiothorac Surg. 2014;45(1):108–13. doi: 10.1093/ejcts/ezt275 . 23711463

[25] Lu X, Huang X, Xue M, Zhong Z, Wang R, Zhang W, et al. Prognostic significance of increased preoperative red cell distribution width (RDW) and changes in RDW for colorectal cancer. Cancer Med. 2023;12(12):13361–73. doi: 10.1002/cam4.6036 . PubMed Central PMCID: PMC10315724. 37143237 PMC10315724

[26] Pedrazzani C, Tripepi M, Turri G, Fernandes E, Scotton G, Conci S, et al. Prognostic value of red cell distribution width (RDW) in colorectal cancer. Results from a single-center cohort on 591 patients. Sci Rep. 2020;10(1):1072. doi: 10.1038/s41598-020-57721-4 . PubMed Central PMCID: PMC6978334. 31974409 PMC6978334

[27] Abrahan LLt, Ramos JDA, Cunanan EL, Tiongson MDA, Punzalan FER. Red Cell Distribution Width and Mortality in Patients With Acute Coronary Syndrome: A Meta-Analysis on Prognosis. Cardiol Res. 2018;9(3):144–52. doi: 10.14740/cr732w . PubMed Central PMCID: PMC5997444. 29904449 PMC5997444

[28] Lippi G, Targher G, Montagnana M, Salvagno GL, Zoppini G, Guidi GC. Relation between red blood cell distribution width and inflammatory biomarkers in a large cohort of unselected outpatients. Arch Pathol Lab Med. 2009;133(4):628–32. doi: 10.5858/133.4.628 . 19391664

[29] Lu F, Pan S, Qi Y, Li X, Wang J. The Clinical Application Value of RDW, CA153, and MPV in Breast Cancer. Clin Lab. 2021;67(2):10.7754/Clin.Lab.2020.200507. doi: 10.7754/Clin.Lab.2020.200507 . 33616344

[30] Okuturlar Y, Gunaldi M, Tiken EE, Oztosun B, Inan YO, Ercan T, et al. Utility of peripheral blood parameters in predicting breast cancer risk. Asian Pac J Cancer Prev. 2015;16(6):2409–12. doi: 10.7314/apjcp.2015.16.6.2409 . 25824773

[31] Dezayee ZMI, Al-Nimer MSM. The Clinical Importance of Measurement of Hematological Indices in the Breast Cancer Survivals: A Comparison Between Premenopausal and Postmenopausal Women. World J Oncol. 2016;7(1):1–4. doi: 10.14740/wjon956e . PubMed Central PMCID: PMC5624681. 28983356 PMC5624681

[32] Sun H, Yin CQ, Liu Q, Wang F, Yuan CH. Clinical Signi fi cance of Routine Blood Test-Associated Inflammatory Index in Breast Cancer Patients. Med Sci Monit. 2017;23:5090–5. doi: 10.12659/MSM.906709 . PubMed Central PMCID: PMC5667583. 29069071 PMC5667583

[33] Akturk OM, Yildirim D, Cakir M, Vardar YM, Erozgen F, Akinci M. Is there a threshold for red cell distribution width to predict malignancy in breast masses? Niger J Clin Pract. 2022;25(3):349–53. doi: 10.4103/njcp.njcp_1583_21 . 35295059

[34] Riedl J, Posch F, Konigsbrugge O, Lotsch F, Reitter EM, Eigenbauer E, et al. Red cell distribution width and other red blood cell parameters in patients with cancer: association with risk of venous thromboembolism and mortality. PLoS One. 2014;9(10):e111440. doi: 10.1371/journal.pone.0111440 . PubMed Central PMCID: PMC4210186. 25347577 PMC4210186

[35] Yao D, Wang Z, Cai H, Li Y, Li B. Relationship between red cell distribution width and prognosis in patients with breast cancer after operation: a retrospective cohort study. Biosci Rep. 2019;39(7):BSR20190740. doi: 10.1042/BSR20190740 . PubMed Central PMCID: PMC6629944. 31262969 PMC6629944

[36] Zou H, Liu SH, Yang R, Wu XJ, Cao YP, Huang HF. Combination of Neutrophil-to-Lymphocyte Ratio and Red Cell Distribution Width With Serum Tumor Markers for the Differential Diagnosis of Breast Cancer and its Association With Pathological Features and Molecular Types. Clin Breast Cancer. 2022;22(4):e526–e35. doi: 10.1016/j.clbc.2021.11.014 . 34963613

[37] Lee HS, Jung EJ, Kim JM, Kim JY, Kim JR, Kim TH, et al. The usefulness of red blood cell distribution width and its ratio with platelet count in breast cancer after surgery and adjuvant treatment: a retrospective study. Gland Surg. 2022;11(12):1864–73. doi: 10.21037/gs-22-410 . PubMed Central PMCID: PMC9840999. 36654946 PMC9840999

[38] Chen CC, Tang WH, Wu CC, Lee TL, Tsai IT, Hsuan CF, et al. Pretreatment Circulating Albumin, Platelet, and RDW-SD Associated with Worse Disease-Free Survival in Patients with Breast Cancer. Breast cancer (Dove Medical Press). 2024;16:23–39. doi: 10.2147/BCTT.S443292 . PubMed Central PMCID: PMC10799625. 38250195 PMC10799625

[39] Yao M, Liu Y, Jin H, Liu X, Lv K, Wei H, et al. Prognostic value of preoperative inflammatory markers in Chinese patients with breast cancer. Onco Targets Ther. 2014;7:1743–52. doi: 10.2147/OTT.S69657 . PubMed Central PMCID: PMC4196795. 25328407 PMC4196795

