# Supplementary figures and images for: Association between red cell distribution width and all-cause mortality in patients with breast cancer: A retrospective analysis using MIMIC-IV 2.0
Source: PLoS One. 2024 May 15;19(5):e0302414. doi: 10.1371/journal.pone.0302414 (PMC11095716; doi:10.1371/journal.pone.0302414)

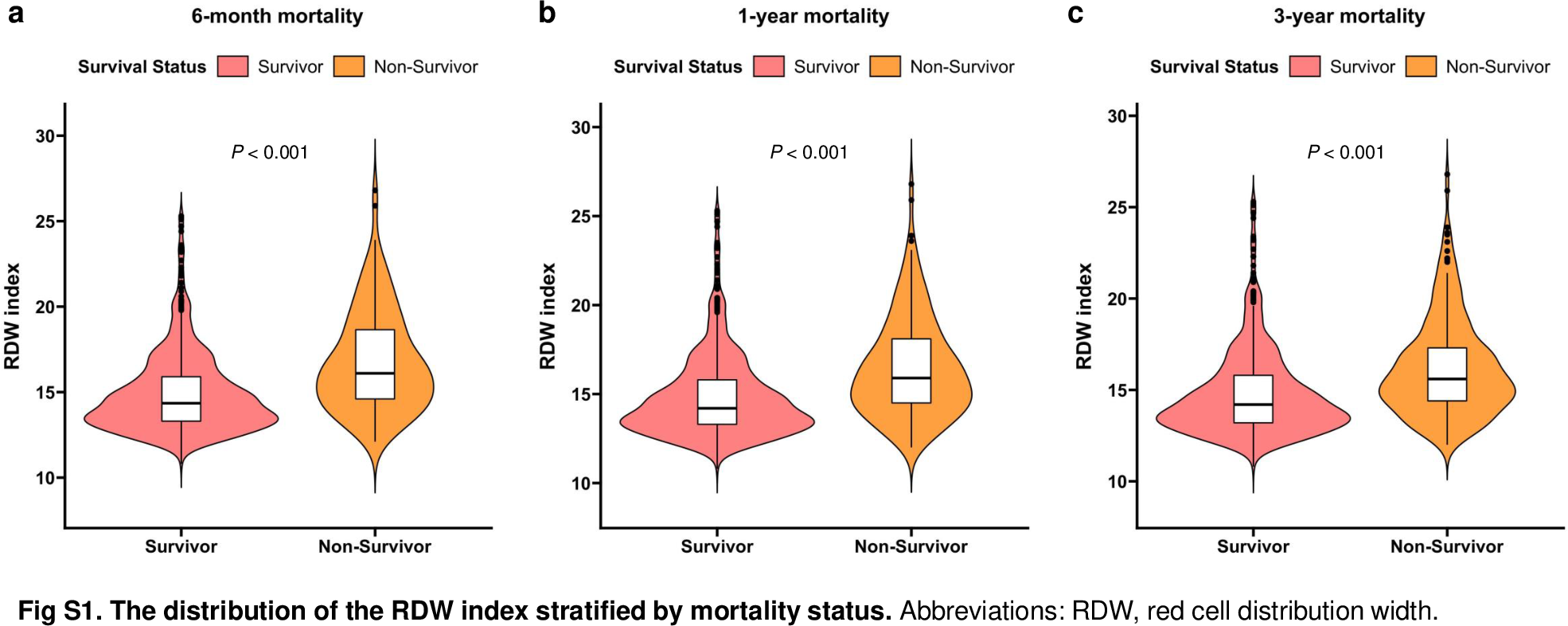

Supplement: S1 Fig — Abbreviations: RDW, red cell distribution width. (TIF) [file pone.0302414.s001.tif]

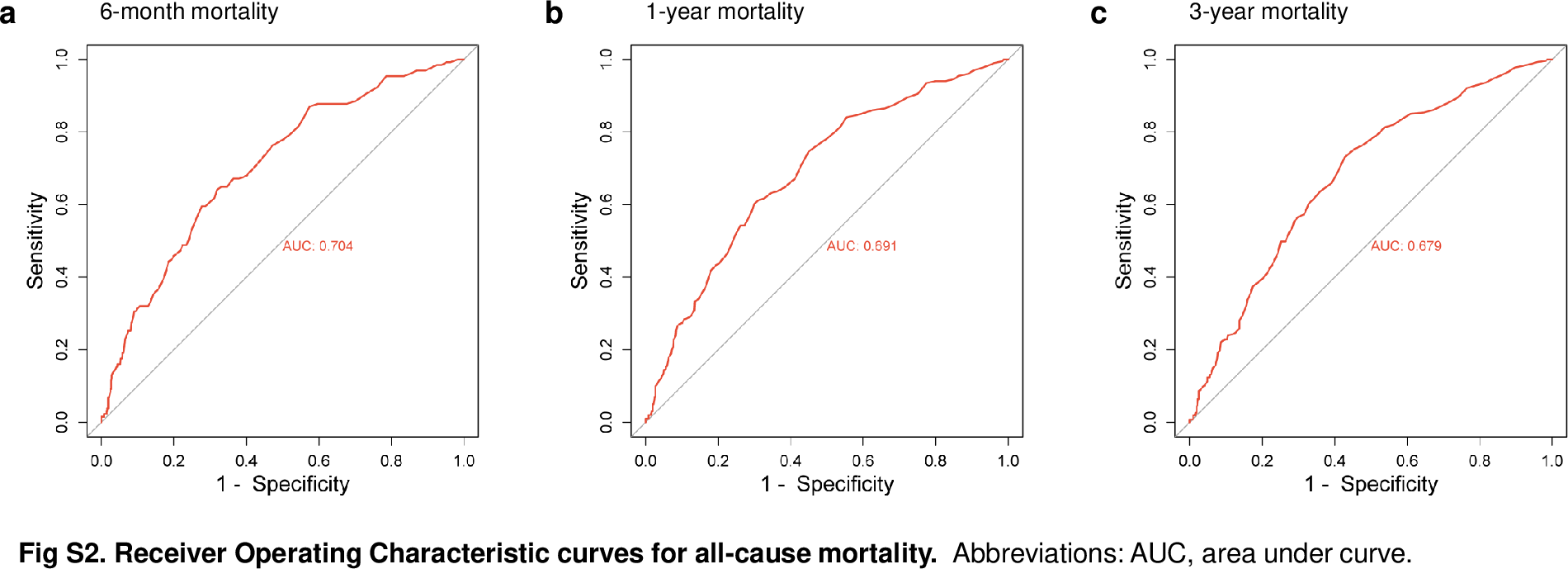

Supplement: S2 Fig — Abbreviations: AUC, area under curve. (TIF) [file pone.0302414.s002.tif]
