# Supplementary material for: Association between red cell distribution width and all-cause mortality in patients with breast cancer: A retrospective analysis using MIMIC-IV 2.0
Source: PLoS One. 2024 May 15;19(5):e0302414. doi: 10.1371/journal.pone.0302414 (PMC11095716; doi:10.1371/journal.pone.0302414)
Supplement: S1 Table — (DOCX) [file pone.0302414.s003.docx]

**Table S1** Missing number for risk variables and outcome variables

| Risk Variables | Missing number (%) |
| --- | --- |
| Age (years) | 0 |
| BMI (kg/m^2^) | 0 |
| Race, white | 0 |
| Medication  Radiation | 0  0 |
| Breast surgery | 0 |
| Comorbidities |  |
| Myocardial infarction | 0 |
| Congestive heart failure | 0 |
| Peripheral vascular disease | 0 |
| Cerebrovascular disease | 0 |
| Chronic pulmonary disease | 0 |
| Rheumatic disease | 0 |
| Mild liver disease  Diabetes without CC  Diabetes with CC  Renal disease  Severe liver disease  Metastatic solid tumor | 0  0  0  0  0  0 |
| Laboratory tests |  |
| AG, mmol/L  Bicarbonate, mmol/L  WBC, ×10^9^/L  RBC, ×10^12^/L  PLT, ×10^9^/L  Hb, g/dL  HCT, %  Chloride, mmol/L  Calcium, mmol/L  Potassium, mmol/L  Sodium, mmol/L  GLU, mg/dL  Creatinine, mg/dL  BUN, mg/dL  MCH, pg  MCHC, % | 114 (12.14%)  114 (12.14%)  0  0  0  0  112 (11.93%)  208 (22.15%)  111 (11.82%)  112 (11.93%)  117 (12.46%)  111 (11.82%)  112 (11.93%)  0  0 |
| MCV, fL | 0 |

Abbreviation: BMI, body mass index; CC; complications or comorbidities; AG, anion gap; WBC, white blood cell; RBC, red blood cell; PLT, Platelet; Hb, Hemoglobin; HCT, hematocrit; GLU, Glucose; BUN, blood urea nitrogen; MCH, mean corpuscular hemoglobin; MCHC, mean corpuscular hemoglobin concentration; MCV, mean corpuscular volume.
